# Patient experiences of participation in a radical thoracic surgical trial: findings from the Mesothelioma and Radical Surgery Trial 2 (MARS 2)

**DOI:** 10.1186/s13063-019-3692-x

**Published:** 2019-10-18

**Authors:** Clare Warnock, Karen Lord, Bethany Taylor, Angela Tod

**Affiliations:** 1 Specialist Cancer Services, Weston Park Hospital, Sheffield Teaching Hospital NHS Foundation Trust, Whitham Road, Sheffield, S10 2SJ England; 2 Glenfield Hospital, Mesothelioma UK/University Hospitals of Leicester NHS Trust, Groby Rd, Leicester, LE3 9QP England; 3 School of Nursing and Midwifery, The University of Sheffield, Barber House Annex, 3a Clarkehouse Rd, Sheffield, S10 2LA England

**Keywords:** Mesothelioma, Clinical trials, Surgery, Chemotherapy, Randomisation

## Abstract

**Background:**

The Mesothelioma and Radical Surgery Trial (MARS 2) aims to evaluate a surgical procedure by comparing chemotherapy and surgery against chemotherapy alone. The pilot study for MARS 2 evaluated the viability of recruitment. Challenges have been reported in conducting clinical research into thoracic surgical treatments and evidence is required to improve our understanding of patient experiences of trial procedures, trial treatments and the factors that influence participation.

**Methods:**

This longitudinal qualitative study was nested within the MARS 2 pilot. Semi-structured telephone interviews were conducted with 15 participants in the MARS 2 trial. Interviews were conducted post-randomisation, post-surgery (surgery arm) and at 6 and 12 months. Altogether, 41 interviews were carried out. The data were analysed using framework techniques.

**Results:**

Challenges were identified regarding the volume and complexity of information given to participants, and their understanding of clinical equipoise and randomisation. Factors influencing participation included having an opportunity to undergo surgery, a self-assessment of their ability to cope with trial treatments, maintaining a positive approach and altruism. Obstacles included the logistics of traveling for treatment in an unfamiliar setting. Negative consequences of trial participation included increased uncertainty amplified by multiple care providers and unclear transition arrangements after the trial.

**Conclusions:**

Participants’ descriptions provided insights that have implications for care for mesothelioma trial patients. The need for healthcare staff to be alert to the potential for misunderstanding, particularly when presenting treatment options, was identified. Patients perceived and derived benefits from taking part in the trial but experienced some negative consequences. These should be anticipated and managed proactively.

**Trial registration:**

ClinicalTrials.gov, NCT02040272. Registered on 20 January 2014.

## Background

Malignant pleural mesothelioma (MPM) is a cancer of the lining of the chest wall and lung. Its aetiology lies in asbestos inhalation and exposure. Incidence is continuing to increase internationally, and, with over 2500 people diagnosed each year, the UK has the highest incidence of mesothelioma in the world [1]. MPM mortality remains high. In the UK, half of patients die within 8.5 months of diagnosis [1]. Chemotherapy is an established treatment for MPM, but response rates are variable [2]. Surgical procedures for MPM may have a valuable role in future treatments [3, 4]. However, access to such treatments varies and there is a need for evidence of the effectiveness and acceptability of surgical interventions for MPM [3–5].

Very little robust, randomised controlled trial evidence exists for MPM surgery, with many studies being observational in nature [4, 6, 7]. As with other cancer treatments, challenges have been reported in conducting clinical research into thoracic surgical treatments, including the reluctance of patients to accept randomisation, a fear of being allocated to the placebo arm, a lack of information and support, restrictive trial regulations, achieving and demonstrating quality control in the surgery, slow recruitment processes and difficulties in presenting trial arm options neutrally [5, 8, 9]. To respond to these challenges, evidence is required to aid our understanding of patient experiences of surgical interventions and trials for MPM, and of the patient motivation to participate.

The current standard treatment for MPM is chemotherapy using the drugs cisplatin and pemetrexed. Mesothelioma and Radical Surgery 2 (MARS 2) is a randomised trial that seeks to compare standard chemotherapy alone with a surgical intervention and standard chemotherapy. The surgical intervention in the trial is extended pleurectomy decortication (EPD), which involves the removal of any visible mesothelioma, the hardened and thickened outer layer of the surface of the lung (decortication) and the lung covering (pleura). Depending on the extent of the disease, all or some of the pericardium and diaphragm may also be removed.

The pilot study for MARS 2 evaluated the potential to recruit to the trial. The primary endpoint was the ability to randomise 50 patients within the first 24 months or the ability to recruit 25 patients in any 6-month period. The pilot stage of MARS 2 is now complete, and MARS 2 has rolled out to a full trial.

The focus of this paper is the qualitative sub-study (QSS) within MARS 2. The QSS was embedded in the pilot stage of MARS 2 and aimed to generate insights into the patient experience of recruitment, consent and randomisation along with the influences and motivations underlying their decisions. The QSS also explored the experience of the MARS 2 treatment interventions and associated care and support needs. This paper presents the findings regarding trial procedures and participation.

## Methods

### Study design

This was an applied health research study that adopted a longitudinal qualitative methodological approach. It was nested within the clinical trial feasibility pilot study for MARS 2.

### Study population

Participants were patients who were taking part in the MARS 2 pilot study, which employed a two-stage unblinded two-arm parallel-design randomised trial. Eligible patients had histological confirmation of MPM such that the disease was confined to one hemi-thorax based on a computerised tomography (CT) assessment. Patients were excluded if they were unable to consent or be randomised, if the disease was not resectable, if they had co-morbidities (respiratory, cardiac, kidney or liver) or if they had a European Cooperative Oncology Group performance status of 2 or more. After two cycles of standard-of-care chemotherapy (platinum and pemetrexed), those whose disease had not progressed beyond surgically resectable limits were randomised to either EPD or no surgery. All patients were then scheduled to receive a further four cycles of standard-of-care chemotherapy. Two surgical centres in England performed the EPD, one in Leicester and the other in Sheffield. A convenience sample of MARS 2 participants were sequentially recruited for the QSS post-randomisation.

### Data collection and analysis

All patients who consented to participate in the MARS 2 pilot study were informed that they may also be invited to take part in the QSS, which was evaluating patient experience. Following randomisation, a member of the QSS research team contacted pilot study participants by telephone to ask if they wished to consider also taking part in the QSS study. Those who agreed to consider participation were given information about the QSS and an opportunity to ask questions during this phone call. Following the telephone conversation, a QSS-specific study participation information sheet and QSS consent form were sent in the post along with a stamped addressed envelope. The first interview with each participant was carried out after the return of the written consent form and confirmation of consent.

Semi-structured telephone interviews were conducted. Participants receiving surgery and chemotherapy were scheduled to be interviewed four times. Interview 1 was post-randomisation but prior to surgery. Subsequent interviews were within 4 weeks after surgery and at 6 and 12 months after the initial interview. Participants receiving chemotherapy alone were interviewed on three occasions. The first was post-randomisation and then at 6 and 12 months following the first interview.

**Fig. 1:**
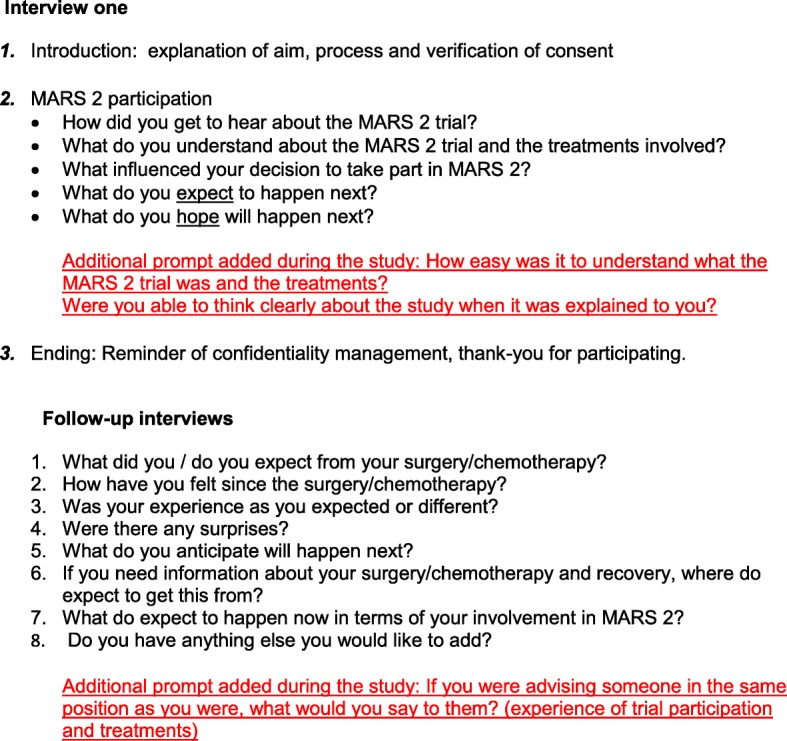
Key questions from the MARS 2 QSS interview topic guide

Interviews were carried out by two researchers (CW and KL) between August 2015 and March 2017. Neither researcher was involved in the wider MARS 2 clinical trial. The interviews ranged in duration from 8 to 45 min and were digitally recorded and transcribed verbatim. A topic guide was developed to keep the interview focused on the participant’s experience of the MARS 2 trial and interventions (Fig. 1). However, the discussion remained flexible to allow for participants to raise issues not anticipated or identified a priori. Previous transcripts for each participant were read prior to their subsequent interviews to enable further discussion of individual themes. The guide was reviewed at a midway point in the data collection, when the team reflected on the emerging findings and additional prompts were added (Fig. 1).

The data were analysed using the framework approach [10]. The transcripts were checked for accuracy and after initial familiarisation with the data, a preliminary thematic framework was developed from the data (CW and KL). The transcripts were independently analysed (AT and BT). The themes were then discussed across the research team and the framework was revised in the light of agreement. The data were then coded and sorted by theme and subtheme to facilitate further analysis and discussion. This process of analysis and discussion was then repeated until a consensus was reached regarding the final thematic framework and findings. This analytic process was carried out at regular points during the data collection and analysis to ensure the emerging findings influenced the data collection and the development of the study findings.

## Results

In total, 16 people were invited to join the QSS. One declined participation as they felt unable to take on additional commitments so that 15 were recruited. Nine participants were receiving chemotherapy and surgery and six receiving chemotherapy alone. Eight participants were from the Leicester centre and seven from the Sheffield centre; 14 were male, age range 59 to 82 (mean 70.13). Out of the potential 54 interviews, 41 were carried out. Seven participants completed all their scheduled interviews. Table 1 is a summary of the interviews completed for each participant.

**Table 1:** Summary of interviews

Study number	Study arm	Interview 1 post-randomisation	Interview 2 post-surgery	Interview 3 6 months’ post-randomisation	Interview 4 12 months’ post-randomisation
1	Surgery	Yes	Yes	No (died)	NA
2	Surgery	Yes	Yes	Yes	No (no reply to contact)
3	Surgery	Yes	Yes	Yes	No (died)
4	Surgery	Yes	Yes	Yes	Yes
5	Chemotherapy	Yes	NA	Yes	Yes
6	Surgery	Yes	No (died)	NA	NA
7	Surgery	Yes	Yes	No (no reply to contact)	No (died)
8	Surgery	Yes	Yes	No (withdrew)	No (died)
9	Chemotherapy	Yes	NA	Yes	Yes
10	Chemotherapy	Yes	NA	Yes	No (died)
11	Surgery	Yes	Yes	Yes	Yes
12	Chemotherapy	Yes	NA	Yes	Yes
13	Chemotherapy	Yes	NA	Yes	Yes
14	Surgery	Yes	Yes	Yes	No (withdrew)
15	Chemotherapy	Yes	NA	Yes	Yes

*NA* not applicable

The analysis identified four main themes, which provide insight into participants’ experiences of the trial procedures and trial participation:

1. learning about the trial
2. deciding to join the trial
3. experience of trial procedures
4. feeling supported during the trial

### Learning about the trial

#### The context of trial information provision

Participants’ descriptions of the events leading up to the trial consultation identified that it took place in a context of new, unexpected and concerning experiences regarding their mesothelioma diagnosis. These included the onset of worrying symptoms, hospital visits, diagnostic tests and investigations, and in most cases, the drainage of litres of fluid from the lung. The majority had received their mesothelioma diagnosis only a few weeks prior to the trial consultation and all had been told that they had a rare incurable cancer associated with a poor prognosis. In addition, they had also learnt that their cancer was an occupational disease caused by exposure to a substance that they may have worked with many years previously, which had legal and financial ramifications. Many described how challenging it had been to assimilate and understand the information and experiences they had gone through before finding out about MARS 2.

*Well, you know, it's in a different field that you've not been experienced in before, and for somebody to turn around and say that you've got this condition and your time is limited, it is very daunting. (Participant 6)*

There were variations in the diagnostic pathways and information provided to each participant prior to their trial consultation but most shared the elements summarised here. One notable difference was the information given at diagnosis about treatment options. Some had been told at the time about the potential for trial treatments, while others had been informed that there was no treatment available:

*[The doctor] said it's inoperable and there was nothing as sure as that, because we were amazed when eventually we came out of hospital and made another appointment to see [a doctor] back at our own local hospital and it was him who suggested the MARS 2 trial. (Participant 9)*

#### Information about treatment options: understanding clinical equipoise

All participants reported feeling informed during recruitment about the treatment options in the trial. However, there was evidence that some had inferred from their trial consultation that surgery could be the preferred treatment from the perspective of the doctor providing the explanation.

*He explained to me that … if it was him personally that had mesothelioma, if surgery was available as well as chemotherapy he would probably go down that, if he had the ability to affect the outcome he would hope for it and probably would want it. (Participant 13)*

*Well, I think he said the surgery's proven to be a bit more … you know, whatever the symptoms are, a bit more controllable or something. (Participant 2)*

However, many also described being told that the trial was being carried out because the optimal treatment for mesothelioma was not known. This included some who recalled that the doctor had inferred a preference.

*It has been explained to me in words of one syllable … that there is no evidence that adding radical surgery will make a massive difference. … To be fair to them, they said we just do not know and that is the reason why we are conducting the trial. (Participant 13)*

In some interviews, it was not possible to identify whether clinical equipoise had been conveyed during the consultation. This was particularly the case when participants described how they felt the surgeon had endorsed surgery when explaining the intention and potential benefits of this treatment. Participants’ descriptions may have reflected inferences they made about the presentation of information by the surgeons or it may have been that the surgeon was not in equipoise.

*He said, well, if you had the surgery … he said two things. he said, it will extend your life and make it easier so if you can get that, that’s fine. (Participant 1)*

*He said, I wouldn't be doing this operation if I didn't think it was going to give you a better time of life. He didn't say what length of life he said better time of life.*

*(Participant 6)*

*He said, even if I take everything I can see, he says, it will still not stop. It will only slow it, you know, he says, but it will give you a bit more time. (Participant 9)*

### Deciding to join the MARS 2 trial

#### Influences on the decision to participate

Reasons given for deciding to participate in the MARS 2 pilot trial were multi-faceted. Where participants were keen to receive surgery, trial participation was a way to achieve this. There was also a perception from some that they might receive enhanced care and support by being on a trial. In many cases, a factor that influenced their decision-making was their own self-assessment of their ability to cope physically with treatment, particularly surgery.

*A lot of people my age have got all sorts wrong with them. I don’t take any medication at all, so it’s got to make a difference. I think when you go to have any sort of operation … then your health, of course, is an important factor in that. (Participant 1)*

Perceived psychological benefits also appeared to influence MARS 2 trial participation. Many spoke of the importance of taking a positive approach to their illness and treatment, and trial participation supported this by providing access to what they felt to be new or up-to-date treatments. In addition, altruism, through improving future treatment, was described as a motivating factor by most participants.

*What I wanted from MARS was that... It has given me some hope, because in the beginning they were … a little bit, “Oh you've only got so long” and all the rest of it, you know what I mean? And I was thinking, “Oh … bugger this for a game of soldiers!” That ... apart from giving other people a chance, that it would also give me a chance, if you understand. (Participant 10)*

#### Potential obstacles to participation

While the participants in this study had all decided to take part in the trial, some had considered not doing so due to the logistics associated with receiving treatment some distance from home. This was both for surgery and chemotherapy, as some had to pass local chemotherapy providers to reach the participating treatment centre. Organising travel and hotel accommodation were challenges for some, and information regarding this was not always readily available.

*And when I saw [the doctor] he said, “Oh, we can help with your expenses and stuff like that.” I went, “Right, okay.” And then I’ve asked a few people … and they don’t really know nothing about it. (Participant 3)*

These logistical issues continued to be a source of concern for some throughout the trial treatments. Having these recognised and acted on by the healthcare team was viewed positively where it had been experienced. An example was scheduling appointments to co-ordinate with train times when there was a long distance to travel.

*I came down to see [the surgeon], and when I spoke to his secretary on the phone, I said, “Look, I need to have an appointment sort of late morning because I’ve got to get the train down,” and that was no bother. She said, “No, that’s fine.” And then my wife and I had a nice train ride down there. That was okay. (Participant 1)*

### Experience of trial procedures

#### Understanding randomisation

There was evidence of variation in the accuracy of participants’ understanding of trial procedures, including randomisation, which is summarised in Table 2. Many used phrases such as “a 50:50 chance” or “could go either way”, which indicates that they were aware that they could get either treatment at randomisation. However, six QSS participants did not fully understand randomisation and the way in which decisions about the treatment they would be receiving were made. Three thought a doctor made the decision based on what was best for the patient. Two thought a computer was given information that helped to select the most appropriate treatment.

*They put all the results … into a computer and then the computer spits out a name. … “I don’t want her. Yes, we’ll have him.” ... Presumably there’s criteria that it has to meet and obviously, because I had responded to the treatment and that’s why I got picked for the surgery. (Participant 11)*

**Table 2:** Understanding of randomisation along with treatment preferences and outcomes

Participant number	Treatment preference	Randomisation outcome	Understanding of randomisation
1	Surgery	Surgery	Good
2	Surgery	Surgery	Good
3	Surgery	Surgery	Not clear. Participant initially described that he could have received either treatment but later in the interview stated that he had been randomised to surgery as the chemotherapy was not working for him.
4	Surgery	Surgery	Good
5	Chemotherapy	Chemotherapy	Good
6	No preference	Surgery	Poor. Patient thought that doctors make the decision and that the trial is comparing types of surgery
7	No preference	Surgery	Not clear from discussion
8	No preference	Surgery	Poor. Patient thought that doctors make the decision
9	Surgery	Chemotherapy	Good
10	Surgery	Chemotherapy	Good
11	Chemotherapy	Surgery	Poor. Patient thought that a computer makes the decision by assessing their individual situation from the information provided
12	Chemotherapy	Chemotherapy	Poor. Patient thought that a computer makes the decision by assessing their individual situation from the information provided
13	Surgery	Chemotherapy	Good
14	Chemotherapy	Surgery	Poor. Patient thought that doctors make the decision and that the trial is comparing types of surgery
15	No preference	Chemotherapy	Good

#### Treatment preferences and randomisation

Participants were asked if they had a treatment preference prior to randomisation. Four declared that they had no preference while seven stated they would have preferred surgery. Two different explanations were given for this. The first was a belief that surgery was inherently a more effective treatment as it physically removed the cancer. The second was a desire to receive all the treatment that was available.

*I felt that if I can use this term “the full loaf” if you like, the whole loaf was really a process of receiving both aspects—chemotherapy and the radical surgery.*

*(Participant 13)*

Four participants declared a preference for chemotherapy, which seemed to be based on concerns about the physical challenges associated with surgery.

*I still consider myself quite fit and I thought to myself maybe if I had that op it could flatten me like and put me out for months and months. So, I wasn’t too upset, put it that way. … If [surgery] had been offered to me, I would have taken it, but secretly I was glad that it wasn’t. (Participant 5)*

For some, the waiting time between consenting to trial participation and the point of randomisation was a period of sustained anxiety.

Interviewer: “Were you aware that you might not have got into that treatment arm”?

The reply was:

*Oh, yes, of course I was, all the time. I was wanting the operation from the beginning and I knew that it was random on a computer and that’s what panicked me. … I don’t know whether chemotherapy works or the other type works, but what I’m saying is I knew my best way would be an operation. … I was worried I might not get the randomisation and get the operation. (Participant 4)*

#### Treatment preferences and randomisation outcomes

Five participants did not get their preferred treatment choice (Table 2). Those who did not get surgery had concerns that chemotherapy might not be as effective as surgery. They also described their disappointment in contrast to their earlier optimism of getting through to randomisation, which meant they had been told their mesothelioma was operable and then informed they were not going to have the operation.

*I sort of got my hopes built up. ... I had a scan, blood tests and then the breathing tests and everything, and the surgeon said that I would be an ideal candidate for it. So, I was sort of upbeat for that … and then it came up with the chemo. … Then, to be perfectly honest with you, I was a little bit, “Oh”. You know what I mean? It was like someone putting a pin in a balloon. Not with it going bang but deflated. (Participant 10)*

The participants who had wanted chemotherapy but had been randomised to surgery responded with stoicism or managed to change their perspective on it

*[I] sort of wished I hadn’t got to go through it, but you take what is given and offered.*

*(Participant 11)*

*Very hesitant. I was still very dubious. … But I think it’s the best way for me to go actually. … I got my head round it and with [my wife] being registered disabled, I look after her as well and I think it’ll gee me on to get things done so I can carry on looking after her. (Participant 14)*

### Feeling supported during the trial

Overall, the care provided during the MARS 2 pilot study recruitment and treatment delivery was described in a positive light. This positive perspective was underpinned by an appreciation that treatment was available and was reinforced by positive feelings towards the National Health Service in general. Other factors relating to care were also praised, such as the behaviours and attitudes of staff towards them as individuals.

*I can't fault the hospital or anything like that. … The nurses and the surgeon and everything like that was good. The people in the CT scan and the X-ray department were brilliant. So I can't, you know, I can't fault any of that. (Participant 10)*

All participants reported feeling informed about the mesothelioma treatment options in the trial, felt they had been given opportunities to discuss them and said that they had not felt under pressure to participate. Many praised the depth and quality of the verbal information that they had received about the trial. Being shown scans and computer images were described as particularly helpful in supporting their understanding of their diagnosis and the surgical procedures, as was the amount of time that staff had taken to give them information and explanations. However, some found the language and detail provided in the written information confusing. For example,

*We tend to get bombarded with paperwork and booklets, and I’ve tried to read them all and some of it makes sense and some of it’s way over my head. … Sometimes understanding the expressions that they use and the descriptions of various things. … Sometimes it does seem an awful lot of stuff to take in. … Not all of it will apply to all people. It’s sometimes a bit confusing sorting out the exact very important bits.*

*(Participant 4)*

Receiving information from more than one team member using a different approach or language was described as one way to make the trial more accessible.

*I went first to see [the surgeon] and there were a nurse there. [The nurse] gave me more literature, but she told me in our term of speaking exactly what it was. So it were down to her … who broke it into my language for me, to understand fully what was going on.*

*(Participant 14)*

Participants indicated that information regarding the trial was in plentiful supply. However, some gaps in the information and support regarding the trial processes and procedures were described in the interviews. These included:

- Clear plans regarding the scheduling of chemotherapy and surgery post-randomisation: For some, there was a period when they did not know what the plans were. Others recalled being given very little notice, for example, being informed of the date a few days before admission.
- Post-trial plans including what will happen after the trial, who will be responsible for their ongoing treatment, who to contact for advice, and future treatments that might be needed or available.

#### Uncertainty and trial participation

There was evidence of fragmented care between the different treatment and service providers, for example, the chemotherapy centre not being aware of the potential date for the resumption of treatment post-operatively. This created a sense of uncertainty and added to participants’ anxiety.

*We had to phone up. And she didn't know nothing about the trial. I said, “Oh, do you know if I'm coming?” “You know more than me,” she said, “I haven't heard nothing yet.” Anyway, I think because it's a new trial, maybe they're sorting things out and that.*

*(Participant 2)*

The end of treatment and the trial were also times that triggered uncertainty among participants. Some found these trial transitions difficult because they were losing contact with healthcare staff with whom they had built a relationship. This appeared to be exacerbated by the absence of a clear plan for ongoing care and support during the post-treatment recovery phase as well as ongoing disease surveillance as they continued to live with mesothelioma.

*I'm not saying that they're not bothered about you. … It just seems they pass you on from one to another and you don't get the same, like, personal attention, sort of thing. It’s not as though they’re doing anything personal, but you don't get the same feedback, the information. They seem to know more about you if you're seeing somebody all the time.*

*(Participant 9)*

Participant: “There were times when there was a long gap with apparently nothing happening. … It was a long time with no clinics. … It seemed a long time, in fact.”

Interviewer: “Would you have preferred someone to have contacted you?”

Participant: “That may well have eased things knowing that you were not forgotten.” (Participant 12)

## Discussion

This longitudinal QSS has generated information about the experiences of patients going through a multi-step clinical trial for mesothelioma. It reveals their motivations to take part and their experiences of trial procedures. It provides insight into why recruitment to previous thoracic surgical trials may have been challenging [7–9].

The study identified considerable challenges in presenting information to patients considering trial participation due to the timing, volume and complexity of information. From the perspective of some participants, an understanding of equipoise was not achieved. Without direct observation of trial communication in clinics, it is not possible to know if what was reported by participants was actually said or was a misunderstanding by the patient. Either way, this finding highlights that staff involved in recruitment need to be aware of the potential for their words to be misinterpreted by patients. This is particularly important, as some participants were subsequently randomised to receiving a treatment that they felt was perceived to be less effective by healthcare staff. The main MARS 2 trial, which is now in progress, contains an embedded Quintet Recruitment Intervention [11], one aspect of which is to audio-record consultations when the MARS 2 study is explained and to interview patients afterwards, so that this issue can be explored further and addressed.

Participants’ understanding of randomisation was also found to be variable. Previous studies have observed that the term “randomisation” and the concept of random allocation is often unknown to patients prior to being offered trial participation [12]. In addition, patients sometimes assume that individual characteristics, or the decision of a doctor, are the reason for their allocation to a particular treatment arm [12, 13]. The findings from the MARS 2 pilot QSS also raise the possibility that trial procedures may contribute to difficulties with understanding complex concepts such as randomisation. In MARS 2, prior to randomisation, the surgeon reviewed a CT scan to determine whether the participant was still eligible for surgery. The data indicate this could have been misinterpreted as the doctor deciding whether surgery was the right treatment for them. Working with patients to co-produce information that communicates trial procedures and processes effectively may be a useful approach to meeting this challenge. A co-production approach could help to identify language and phrases that have less scope for misunderstanding.

Most participants of the QSS held preferences for a particular treatment. The time leading up to randomisation was experienced as a period of anxious uncertainty, as they waited to find out if they were to receive their preferred intervention. Participants’ descriptions of their experiences suggest that consultations regarding randomisation outcomes should be recognised as moments when significant information is given. When the allocation differs from the patient’s preferred outcome, it may be beneficial to approach it as breaking bad news and managed in the same way.

Agreement to enter the trial was associated with additional pressures for study participants. Acknowledging the negative implications of trial participation in initial discussions may help to prepare participants for this. While a degree of uncertainty is to be expected, our study revealed that it was exacerbated when there was poor coordination or communication between treatment providers. Uncertainty was common when patients moved to post-treatment recovery and surveillance. Indeed, some accounts of this period provided a stark comparison between the intensity of support at recruitment compared to when patients were exiting the trial. The potential for patients to have negative experiences when leaving a trial has been identified in previous research, with some patients describing how they felt abandoned or “set loose” as they lose the support that accompanies trial participation [14]. Employing a care coordinator or navigator may provide a solution to these problems. Care navigators track the patient’s progress along the trial and treatment pathway, keep in touch with the patient and facilitate communication between the different service providers, which has led to positive patient outcomes in other cancer settings [15]. This may be a useful role to consider in clinical trials care. In addition, an exit consultation in which future treatment and surveillance plans are outlined and which implements elements of the Macmillan Cancer Support Recovery Package [16] may help to ease uncertainty after a clinical trial and bridge the gaps between secondary and primary care.

The main strength of our study is the use of longitudinal qualitative data collection methods to explore the reasons behind participants’ decisions to participate in a clinical trial and their understanding and experience of trial procedures. The sample size is relatively small, but the longitudinal methods provided opportunities to revisit themes and subjects arising from earlier interviews and capture participants’ experience during the trial, from early involvement when the focus is on the trial procedures, randomisation and the decision to participate, to later stages as participants leave the trial. While sampling in qualitative methods is not intended to be representative, participants were from a wide geographical area. The findings are limited to those who were mesothelioma patients and who agreed to participate in the MARS 2 clinical trial; therefore, saturation may not have been achieved. However, an in-depth understanding of patients’ experience across time was generated. The experiences of those who declined participation pre- or post-randomisation would provide additional and potentially different insights into the subjects explored.

## Conclusion

The study provides insights into the challenges facing patients in absorbing and understanding the volume of complex information associated with trial participation. It highlights the importance of healthcare staff being alert to the potential for misunderstanding, particularly when presenting treatment options. Patients perceived and derived benefits from taking part in the trial, but negative consequences, such as uncertainty regarding treatment plans and transition arrangements at trial completion, should be anticipated and managed proactively.

## Data Availability

The data used and analysed are available from the corresponding author on reasonable request.

## Abbreviations

CT: Computerised tomography
EPD: Extended pleurectomy decortication
MARS 2: Mesothelioma and Radical Surgery Trial
MPM: Malignant pleural mesothelioma
QSS: Qualitative sub-study
